# Absence of Epstein-Barr virus DNA in anti-citrullinated protein antibody-expressing B cells of patients with rheumatoid arthritis

**DOI:** 10.1186/s13075-022-02919-2

**Published:** 2022-10-13

**Authors:** Sanne Kroos, Arieke S. B. Kampstra, René E. M. Toes, Linda M. Slot, Hans U. Scherer

**Affiliations:** Department of Rheumatology, Leiden University Medical Center, Albinusdreef 2, 2333 ZA Leiden, The Netherlands

## Abstract

**Objective:**

Rheumatoid arthritis (RA) is characterized by the presence of disease-specific autoreactive B cell responses, in particular those generating anti-citrullinated protein antibodies (ACPA). For many years, Epstein-Barr virus (EBV) has been implicated in disease pathogenesis, possibly by facilitating the development and persistence of autoreactive B cells. To test this hypothesis, the presence of EBV episomes in ACPA-expressing B cells was analyzed.

**Methods:**

ACPA-expressing B cells derived from peripheral blood (PB) of seven EBV-seropositive RA patients, and synovial fluid (SF) of one additional EBV-seropositive RA patient, were isolated by flow cytometry. PB cells were expanded for 11–12 days, after which supernatant was harvested and analyzed for cyclic citrullinated-peptide (CCP)2 reactivity. SF cells were isolated directly in a lysis buffer. DNA was isolated and qPCR reactions were performed to determine the EBV status of the cells. EBV-immortalized B cell lymphoblastoid-cell lines (EBV blasts) served as standardized controls.

**Results:**

Two hundred ninety-six PB and 60 SF ACPA-expressing B cells were isolated and divided over 16 and 3 pools containing 10–20 cells, respectively. Supernatants of all 16 cultured PB pools contained CCP2-Ig. DNA of all pools was used for qPCR analysis. While EBV-blast analysis showed sensitivity to detect EBV DNA in single B cells, no EBV DNA was detected in any of the ACPA-expressing B cell pools.

**Conclusion:**

ACPA-expressing B cells are not enriched for EBV-DNA-containing clones. These results do not support the hypothesis that EBV infection of autoreactive B cells causes or maintains autoreactive B cell populations in RA. Instead, other mechanisms might explain the association between positive EBV serology and RA.

**Supplementary Information:**

The online version contains supplementary material available at 10.1186/s13075-022-02919-2.

## Introduction

Rheumatoid arthritis (RA) is an autoimmune disease characterized by synovial tissue inflammation resulting in swollen, painful joints and bone erosions. A hallmark of RA is the presence of autoantibodies against posttranslational modifications, in particular anti-citrullinated protein antibodies (ACPA). Whether these autoantibodies are directly pathogenic is still under debate, but multiple features of the ACPA response and the clinical response of patients to therapeutic depletion of CD20^+^ B cells strongly indicate that ACPA-expressing B cells are intimately involved in disease pathogenesis [1].

The initial cause for RA development is unknown. However, an interplay between genetic predisposition and environmental factors is considered essential in this context [1]. For more than 30 years, an environmental factor suspected to play a role in RA development is Epstein-Barr virus (EBV). EBV infection is highly prevalent in humans, affecting over 90% of individuals worldwide [2]. EBV preferentially infects B cells. After control of the initial infection, the virus latently persists in approximately 1 in 10,000 to 100,000 memory B cells for the lifetime of a given individual [3].

It has been suggested that RA patients show impaired control of EBV infection. RA patients harbor less efficient T cell responses against EBV, increased anti-EBV antibody levels, and an increased number of EBV-infected B cells compared to healthy controls [4]. Whether and how EBV contributes to RA pathogenesis, however, is unclear, and multiple hypotheses have been postulated. One hypothesis proposes that EBV infection allows autoreactive B cells to escape clonal deletion, based on the observation that constitutive latent membrane protein 2A (LMP2A) expression leads to autoreactive B cell activation in transgenic mice [5]. In fact, LMP2A provides continuous survival and proliferation signals by mimicking B cell-receptor activation. Latent membrane protein 1 (LMP1), another viral protein expressed by EBV-infected B cells, is a functional homolog of CD40 and activates downstream pathways independent of B cell interaction with CD154-expressing T cells [6]. Hence, through the expression of LMP2A and LMP1, EBV could stimulate the selection of autoreactive B cells which would otherwise be deleted. In fact, one study reported the expression of EBV proteins in RA synovial tissue plasma cells that bound citrullinated antigens in histological stainings [7].

To test and substantiate this hypothesis, we here employed technology to identify and isolate ACPA-expressing B cells from patients to high purity and screened these cells for EBV positivity. We used methodology capable of detecting EBV episomal DNA in single B cells, based on the presence of DNA encoding EBV’s major tegument protein BNRF1. Nonetheless, we could not detect the enrichment of EBV-positive clones in this autoreactive B cell population. Hence, we consider it unlikely that the associations between EBV and RA are explained by a mechanism by which EBV facilitates autoreactive B cell development directly through persistence in autoreactive clones [4].

## Patients and methods

### Patients

Peripheral blood (PB) or synovial fluid (SF) from eight ACPA-positive RA patients was collected at the outpatient clinic of the Department of Rheumatology at Leiden University Medical Center (LUMC). All RA patients met the 2010 American College of Rheumatology/European League against Rheumatism (ACR/EULAR) criteria for RA at the time of diagnosis. Treatment regimens included csDMARDs, glucocorticoids, and biologic agents. Permission for conduct of the study was given by the ethical review board of LUMC. All donors gave written informed consent. All included patients were determined to be EBV-seropositive by the presence of anti-EBV nuclear antigen (EBNA)-IgG and anti-viral capsid antigen (VCA)-IgG antibodies in their plasma (Table 1).

**Table 1:** Patient characteristics. Values of the most recent CCP2-IgG plasma titer, EBNA-IgG plasma titer, and VCA-IgG plasma titer were determined by diagnostic testing. Of patients one to seven, PB was analyzed and depicted in Figs. 1A–C and 2D. From patient eight, we analyzed SF and the results are depicted in Supplementary Fig. 2. Disease activity was assessed by routine clinical assessment of 28 joints for pain and swelling and subsequent calculation of the DAS28(3v)-ESR

Patient	Age	Sex	Treatment at time of blood collection	CCP2-IgG titer (U/mL)	EBNA-IgG titer (U/mL)	VCA-IgG titer (U/mL)	Isolated ACPA cells per pool	Time since CCP2+ RA diagnosis	DAS28(3v)-ESR
1	65	F	Methotrexate	> 340	26,9	455	6 x 20 cells, 1 x 18 cells	3 years and 3 months	1,97
2	68	M	Methotrexate + hydroxychloroquine	> 313	> 600	742	1 x 20 cells, 1 x 18 cells	2 years and 3 months	1,82
3	79	F	Methotrexate	> 340	161	> 750	1 x 18 cells	25 years	1,97
4	69	F	Prednisone	> 340	40,8	524	2 x 20 cells	24 years	5,11
5	75	F	Etanercept	> 313	238	> 750	2 x 20 cells	27 years	1,97
6	71	F	Leflunomide	> 265	12,1	> 750	1 x 10 cells	5 years	3,40
7	58	M	Methotrexate	> 340	> 600	> 750	1 x 12 cells	4 months	2,16
8	49	F	Methotrexate	> 340	272	> 750	3 x 20 cells	21 years	3,84

### Antigen labeling

Allophycocyanin (APC) or Brilliant Violet 605 (BV605)-labeled streptavidins were conjugated to a biotinylated cyclic citrullinated peptide (CCP)2 and the arginine-control variant CArgP2 was conjugated to phycoerythrin (PE)-labeled extravidin, as described previously [8]. Optimal concentrations of these tetramers for identifying citrullinated antigen-specific B cells were determined by titrations on Ramos 3F3 and Ramos MDL-AID KO cell lines [9].

### ACPA-B cell isolation, culture, and DNA isolation

PBMCs and SFMCs were isolated by Ficoll-Paque gradient centrifugation. SF was treated with hyaluronidase (Sigma-Aldrich) before subjection to Ficoll-Paque. Isolated cells were stained with Live/dead Fixable Violet dead cell-stain kit 451 nm (life technologies), CD3-PB (UCHT1, BD), CD14-PB (M5E2, BD), CD19-APC-Cy7 (Sj25C1, BD), CD20-AF700 (2H7, BioLegend), CD27-PE-Cy7 (M-T271, BD), IgG-BV510 (G18-145, BD), IgD-FITC (IA6-2, BD), CCP2-BV605, CCP2-APC, and CArgP2-PE. Pools of ACPA-expressing B cells (dead cell-staining negative, CD3^−^, CD14^−^, CD19^+^, CCP2^+/+^, CArgP2^−^) were isolated using a FACS Aria III 4L (BD Biosciences) cell sorter at the Flow Cytometry Core Facility of the LUMC. In total, 296 ACPA-expressing B cells were isolated and divided over 16 pools containing 10–20 cells each. As the majority of ACPA-expressing B cells have a CD20^+^ CD27^+^ memory phenotype [8], equal pools of IgG^+^ memory B cells not expressing ACPA (dead cell-staining negative, CD3^−^, CD14^−^, CD19^+^, CD20^+^, CD27^+^, IgG^+^, IgD^−^, CCP2^−/−^) were isolated as controls (Supplementary Fig. 1). PB cells were isolated in a flat-bottom 96-well culture plate containing 10,000 cells/well irradiated mouse fibroblast cells expressing human CD40 ligand (CD40L) in complex B cell-culture medium (IMDM, 8% FCS, 100 U/ml penicillin/streptomycin, 1 ng/ml IL-1β, 500 ng/ml R848, 50 ng/ml IL-21, 0.3 ng/ml TNF-α, 20 μg/ml IgG-depleted apotransferrin, 50 μM β-mercaptoethanol). Cells were kept in culture for 11–12 days at 37°C/5% CO_2_. Thereafter, supernatants were tested in ELISA. Cell pellets were resuspended in 45 μl of PicoPure™ proteinase K buffer (ThermoFisher) and transferred to a 96-well PCR plate. SF cells were isolated and lysed directly without culture. For DNA isolation, 96-well PCR plates containing cells in proteinase K buffer were incubated at 65°C for 3 hours, followed by 95°C for 15 min, and stored at −20°C until further use.

JY cells were obtained from ATCC and cultured in IMDM containing 8% FCS, 100U/ml penicillin/streptomycin, and 2mM GlutaMax.

### CCP2-Ig and total-Ig ELISAs

The presence of CCP2-Ig, CArgP2-Ig, and total-Ig in culture supernatants was assessed by ELISA. For the CCP2 and CArgP2 ELISA, C96 Maxisorp Nunc Immuno plates (Thermo Fisher Scientific) were coated with streptavidin, to which biotinylated CCP2 or CArgP2 peptide was added. For total-Ig, 96 well plates were coated with goat anti-human IgG + IgM + IgA heavy and light chain (Bethyl) capture antibodies followed by a blocking step with PBS/1%BSA/50mM Tris, pH 8.0. For all three ELISAs, samples were tested in a 1:2 or 1:5 dilution. CCP2-Ig, CArgP2-Ig, or total-Ig was detected by HRP-conjugated polyclonal goat anti-human IgG+IgM+IgA heavy and light chain antibodies (Bethyl). Absorbance was measured at 415 nm using ABTS + H_2_O_2_ (Sigma-Aldrich) on an iMark™ Microplate Absorbance Reader (Bio-Rad).

### Probe-based qPCRs

Proteinase K isolated DNA from individual B cell pools was subjected to qPCR using *BNRF1* and Beta-2-Microglobulin (*B2M*)-specific primers in order to determine the presence or absence of EBV DNA and human DNA, respectively. qPCR reactions were performed in duplicates using 2.5 times diluted DNA, Hot StarTaq Master Mix Kit (Qiagen), 300nM forward and reverse primers, and 250nM probe. *B2M* forward: 5′-GGATAGGTGAGGACTATC-3′. *B2M* reverse: 5′-CCTGTGGATGCTAATTAAA-3′'. *B2M* probe: 5′-/5Cy5/AAGTATGTTCTGTCCCATTTGC/3IAbRQSp/-3′. *BNRF1* forward: 5′-GGAACCTGGTCATCCTTTGC-3′. *BNRF1* reverse: 5′-ACGTGCATGGACCGGTTAAT-3′. *BNRF1* probe: 5′-/56-FAM/CGCAGGCACTCGTACTGCTCGCT/3BHQ_1/-3′. Negative controls included H_2_O and CD40L L cells only from the isolation and culture procedure. Positive controls included the standard curve generated from JY cell DNA and wells containing DNA from either 1, 2, 5, 10, 20, 50, or 100 JY cells. qPCR protocol: 15′ 95°C, 40 times 5″ 95°C 15″ 55°C (camera), 15″ 72°C.

## Results

### Isolated and cultured PB B cells are specific for CCP2

**Fig. 1:**
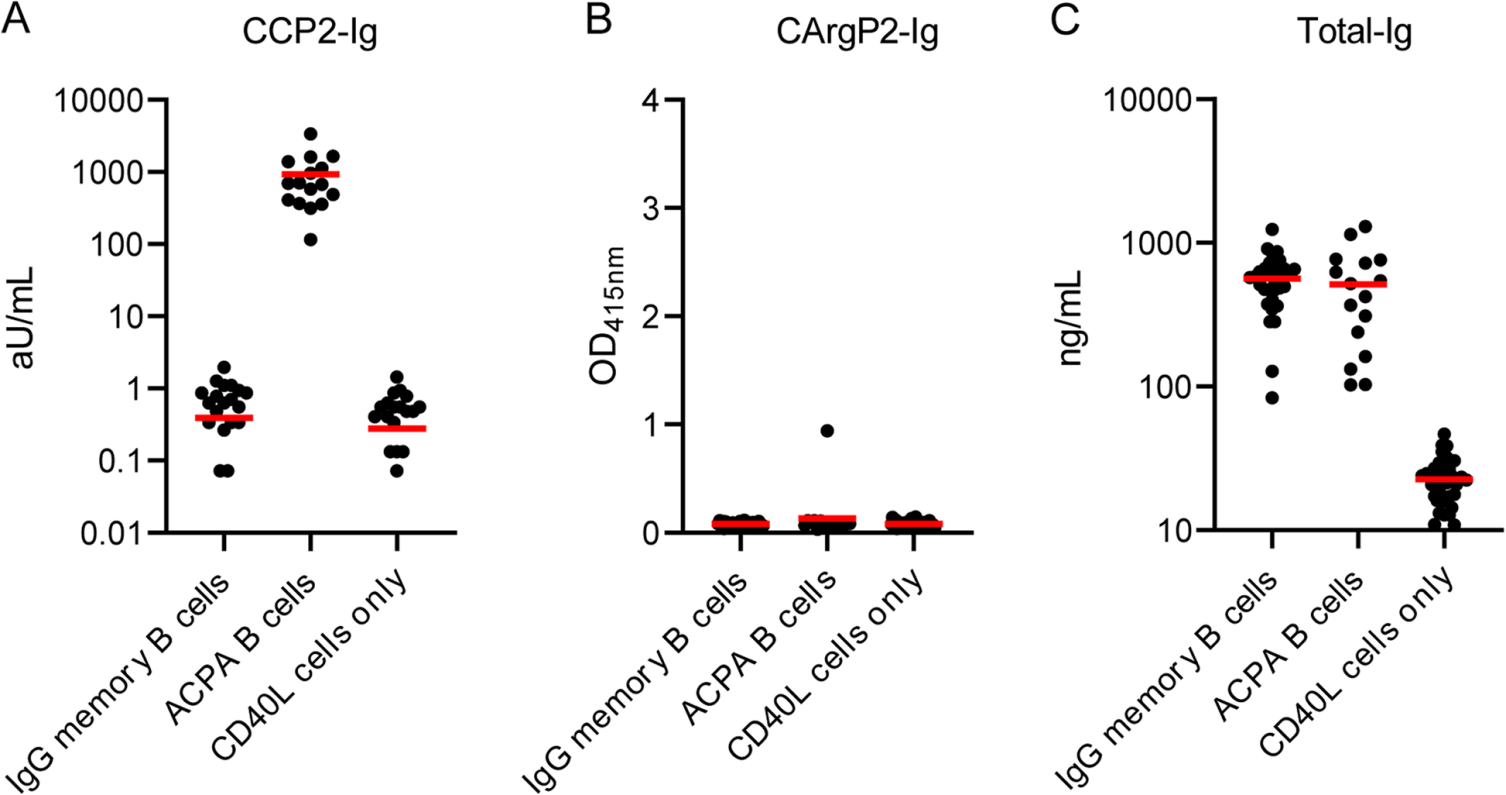
Isolated PB ACPA-expressing B cells are CCP2-reactive and specific. **A** CCP2-Ig ELISA of supernatants of isolated and cultured PB ACPA-expressing B cells. No CCP2-binding was observed in supernatant of control IgG memory B cells and CD40L feeder cells only. **B** CArgP2-Ig ELISA of supernatants of isolated and cultured PB ACPA-expressing B cells. No or low CArgP2-Ig binding was observed. **C** Total-Ig ELISA of supernatants of isolated and cultured control PB IgG memory B cells and of PB ACPA-expressing B cells. No Ig was detected in wells containing CD40L feeder cells only

PB ACPA-expressing B cells from patients with RA (*n*=7) were isolated in pools of 10-20 cells per well to determine their EBV status. As the majority of ACPA-expressing B cells exhibited an IgG memory phenotype, non-ACPA-expressing IgG memory B cells were isolated as controls. Following the culturing period, supernatants were harvested and tested for CCP2 reactivity. Indeed, CCP2-Ig ELISA confirmed specific reactivity towards CCP2 in supernatants obtained from ACPA-expressing B cell pools, whereas the control pools remained negative (Fig. 1A and B). Also, control wells with only CD40L feeder cells harbored no reactivity towards CCP2 (Fig. 1A). ELISA confirmed the production of total Ig by both control IgG memory B cells as well as ACPA-expressing B cells. Wells containing only CD40L feeder cells did not demonstrate detectable Ig production (Fig. 1C).

### *BNRF1* is undetectable in DNA from ACPA-expressing B cells

**Fig. 2:**
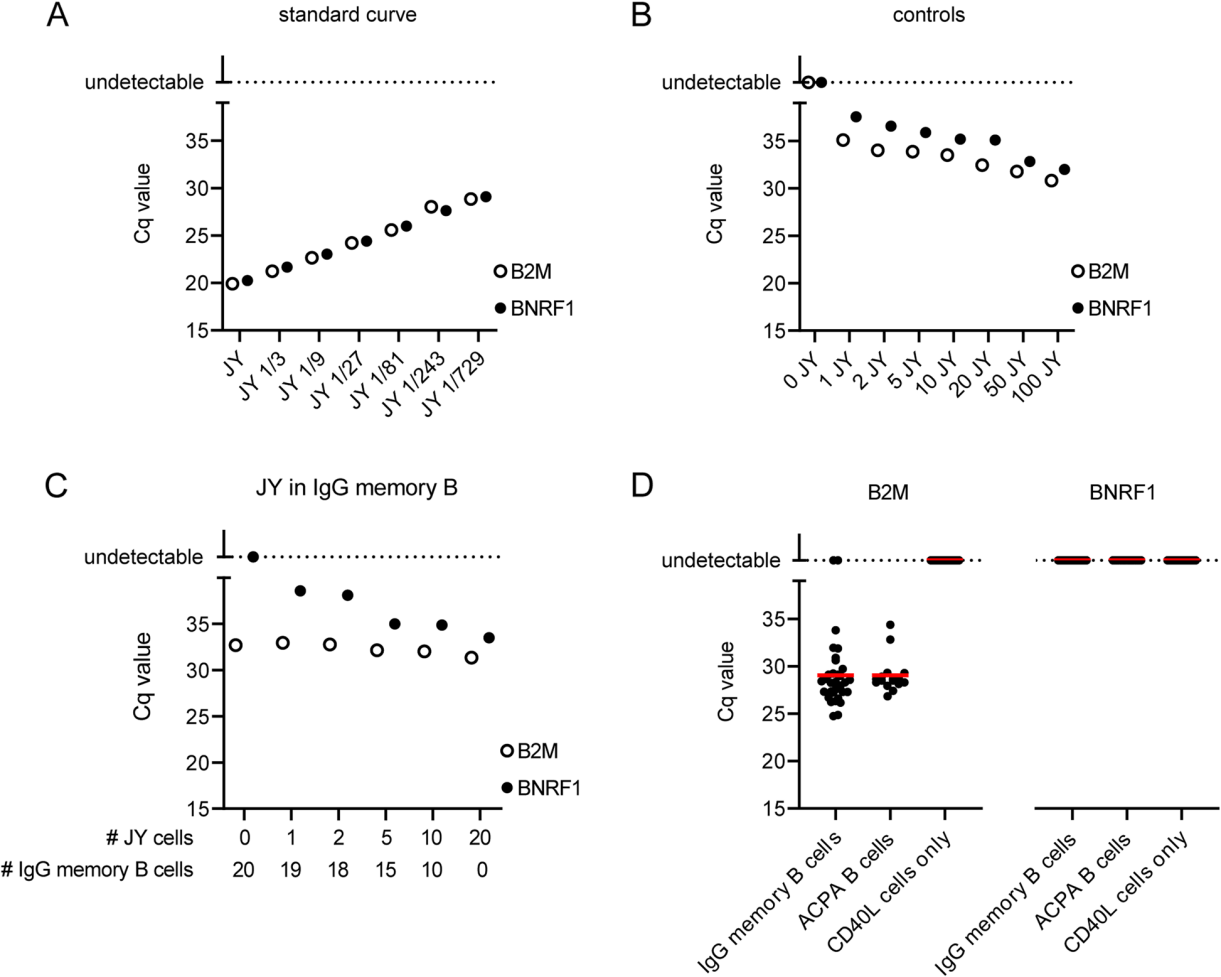
BNRF1 is undetectable in PB antigen-specific ACPA-expressing B cells. **A ***B2M* and *BNRF1* qPCR of serial dilutions of pooled JY cell DNA. 1/3 indicates a 1:3 dilution of DNA from 500,000 JY cells, 1/9 indicates a 1:9 dilution of DNA from 500,000 JY cells, etcetera. **B ***B2M* and *BNRF1* qPCR of specific numbers of JY cells. *BNRF1* was readily detectable in one single JY cell. With increasing cell numbers, the Cq value decreased correspondingly. **C ***B2M* and *BNRF1* qPCR of JY cells titrated into PB IgG memory B cells. **D** Left: *B2M* copies in DNA of PB IgG memory B cells and ACPA-expressing B cells as detected by qPCR. *B2M* was not detected in the DNA of CD40L feeder cells only. Right: no detection of *BNRF1* by qPCR in DNA of PB IgG memory B cells, ACPA-expressing B cells, and CD40L feeder cells only. Each dot in IgG memory B cells represents a pool of 20 isolated and cultured cells, from seven different donors. Each dot in ACPA-expressing B cells represents a pool of 10–20 isolated and cultured cells, from seven different donors. qPCR experiments were performed twice with similar outcomes

To detect EBV infection of isolated B cells, we investigated the presence of EBV DNA in ACPA-expressing B cells using the detection of the *BNRF1* gene by qPCR. The EBV-immortalized B lymphoblastoid-cell line JY served as positive control and allowed to determine the sensitivity of the *BNRF1* gene qPCR. The undiluted standard curve sample was prepared with DNA isolated from 0.5 million JY cells by proteinase K incubation (Fig. 2A). To investigate the sensitivity of the qPCR, 1, 2, 5, 10, 20, 50, or 100 JY cells were directly isolated by FACS into proteinase K buffer. With this approach, *BNRF1* and *B2M* from one single JY cell could be detected. As expected, increasing the number of JY cells decreased the Ct value of the qPCRs (Fig. 2B). Additionally, we titrated 0, 1, 2, 5, 10, and 20 JY cells into 20, 19, 18, 15, 10, and 0 IgG memory B cells, respectively. This indicated that *BNRF1* from JY cells is still detectable when diluted in non-*BNRF1-*carrying DNA (Fig. 2C). Finally, DNA from isolated, ACPA-expressing B cells was subjected to qPCR reactions measuring levels of *B2M* and *BNRF1*. After 40 cycles, *BNRF1* copies in DNA from PB ACPA-expressing B cells remained undetectable, whereas *B2M* copies were readily detectable after, on average, 28–29 cycles, indicating the presence of human DNA but not EBV DNA (Fig. 2D). Furthermore, *BNRF1* copies were undetectable in pools of directly lysed SF ACPA-expressing B cells whereas *B2M* was readily detectable after 32–33 cycles (Supplementary Fig. 2).

## Discussion

Several studies have indicated a role for EBV infection in the development of RA [4]. So far, however, no exact mechanism has been identified for these observations. ACPA-expressing B cells and their antibodies have been proposed to arise due to EBV infection of B cells and their viral proteins shielding autoreactive B cells from clonal deletion [5]. In support of this hypothesis, EBV infection was previously indicated in 70% of ACPA-expressing plasma cells by immunofluorescence staining of EBV viral proteins in the periphery of ectopic lymphoid neogenesis (ELN) tissue in the synovium of RA patients [7]. These observations indicate a role of EBV in, e.g., maintenance of ACPA-expressing cells as, on average, only 1 in every 10–100,000 memory B cells is infected with EBV in donors showing positive EBV serology [3]. To further test and substantiate this hypothesis, we therefore employed our well-established technology to isolate ACPA-expressing B cells from the blood of RA patients (including patients with active disease or low disease activity/remission) and subsequently cultured pools of these primary cells, reasoning that if EBV infection would be a decisive factor in selection and survival of these cells, EBV DNA should be present in most of the clones. Upon confirmation of their antigen-specificity by CCP2 ELISA, DNA of pooled cells was used to determine the presence or absence of viral *BNRF1* DNA to indicate EBV infection. While we could readily confirm the presence of human DNA by detecting *B2M* in all B cell pools, we did not detect *BNRF1*, indicating the absence of EBV viral copies in PB ACPA-expressing B cells. Also, we could not detect *BNRF1* in ACPA-expressing B cells isolated from SF. These results suggest that EBV infection of autoreactive B cells themselves is not a facilitating factor for infected B cells to become or persist as autoreactive clones. This conclusion is supported by a cohort of EBV-seronegative patients transplanted with kidneys from EBV-seropositive donors, resulting in primary EBV infection of transplant recipients. Serum samples taken before and after transplantation revealed no de novo induction of either IgG- or IgM anti-CCP2 antibodies [10]. Together, these data argue against a causative relationship between EBV infection and the development of ACPA-expressing B cells.

Our experimental approach allowed us to detect EBV DNA in as little as one single JY cell. Next to a direct titration of JY cells, we titrated 0, 1, 2, 5, 10, and 20 JY cells into 20, 19, 18, 15, 10, and 0 IgG memory B cells, respectively, showing that *BNRF1* in single JY cells was still detectable when diluted in non-*BNRF1* carrying DNA. Nonetheless, limitations of assay sensitivity need to be considered when interpreting our data. JY cells have been quantified to harbor less than 10 EBV copies per cell [11], which could potentially be even less in individual ACPA-expressing B cells. By culturing, we allowed the isolated B cells to expand and reasoned that expansion of any one cell carrying EBV DNA at the time of isolation would increase EBV copies in the pools and, thereby, the chances of detection by PCR. Still, despite this sensitive approach and although unlikely, we cannot fully exclude that EBV DNA present in very low copy numbers in single, primary ACPA-expressing (memory) B cells could have escaped our detection. Likewise, our study is limited by the very low frequency of ACPA-expressing B cells in patients and, hence, low cell numbers that we could acquire/test. Nonetheless, given a reported almost 10-fold increase in EBV DNA load in PBMCs from RA patients compared to controls [12], we consider it likely that a relevant contributing effect of EBV infection to survival/outgrowth of ACPA-expressing B cells would yield a frequency of EBV-positivity in the antigen-specific B cell population that we would have been able to detect.

Irrespective of the above, the associations between EBV and RA remain intriguing. While our research indicates that EBV does not directly facilitate the development/survival of ACPA-expressing B cells, it remains a possibility that EBV induces other types of autoantibodies relevant in RA such as Rheumatoid Factor [13]. An alternative hypothesis to the direct infection of autoreactive B cells tested here is that autoreactivity in RA could be triggered by molecular mimicry. Rather than EBV causing autoreactive B cells to escape clonal deletion, antibodies directed against EBV antigens could cross-react with self-antigens. Indeed, molecular similarities have been identified between EBV and self-antigens relevant in RA. Also, ACPA-IgG have been reported to react with citrullinated EBV peptides [14–16]. Moreover, molecular mimicry between EBV- and self-antigens has also been indicated in the context of other autoimmune diseases, for example in the development of multiple sclerosis (MS). Recently, molecular mimicry between EBNA1 and glial cell adhesion molecule (GlialCAM), facilitated by post-translational phosphorylation, was reported [17]. In RA, it will be interesting to test whether and to what extent ACPA-IgM expressing B cells show reactivity to citrullinated or otherwise post-translationally modified EBV peptides, and whether recognition of such antigens is involved in initially or continuously triggering of ACPA-expressing B cells.

In conclusion, our results indicate that EBV infection of B cells is by itself not involved in the rise of ACPA-expressing B cells and their escape from immune-tolerance mechanisms. Further research is needed to determine whether EBV infection has another role in RA development.

## Supplementary Information

**Additional file 1: Supplementary figure 1.** Gating strategy of the isolated ACPA-expressing and IgG memory B cell pools.

**Additional file 2: Supplementary figure 2.** BNRF1 is undetectable in SF antigen-specific ACPA-expressing B cells. Left: *B2M* copies in DNA of SF IgG memory B cells and ACPA-expressing B cells as detected by qPCR. Right: no detection of *BNRF1* by qPCR in DNA of SF IgG memory B cells, ACPA-expressing B cells and CD40L feeder cells only. Each dot represents a pool of 20 cells isolated directly into lysis buffer, from one donor. qPCR experiments were performed twice with similar outcomes.

## Data Availability

The datasets used and/or analyzed during the current study are available from the corresponding author on reasonable request.

## Abbreviations

RA: Rheumatoid arthritis
ACPA: Anti-citrullinated protein antibodies
EBV: Epstein-Barr virus
PB: Peripheral blood
SF: Synovial fluid
CCP: Cyclic citrullinated-peptide
LMP2A: Latent membrane protein 2A
LMP1: Latent membrane protein 1
LUMC: Leiden University Medical Center
ACR/EULAR: American College of Rheumatology/European League against Rheumatism
EBNA: EBV nuclear antigen
VCA: Viral capsid antigen
APC: Allophycocyanin
BV605: Brilliant Violet 605
CD40L: CD40 ligand
BNRF1: EBV major tegument protein
B2M: Beta-2-microglobulin
GlialCAM: Glial cell adhesion molecule

## References

[1] Scherer HU, van der Woude D, Toes REM (2022). From risk to chronicity: evolution of autoreactive B cell and antibody responses in rheumatoid arthritis. Nat Rev Rheumatol.

[2] Smatti MK, Al-Sadeq DW, Ali NH, Pintus G, Abou-Saleh H, Nasrallah GK (2018). Epstein-Barr Virus Epidemiology, Serology, and Genetic Variability of LMP-1 Oncogene Among Healthy Population: An Update. Front Oncol.

[3] Laichalk LL, Hochberg D, Babcock GJ, Freeman RB, Thorley-Lawson DA (2002). The Dispersal of Mucosal Memory B Cells: Evidence from Persistent EBV Infection. Immunity..

[4] Balandraud N, Roudier J (2018). Epstein-Barr virus and rheumatoid arthritis. Joint Bone Spine.

[5] Swanson-Mungerson M, Longnecker R (2007). Epstein-Barr virus latent membrane protein 2A and autoimmunity. Trends Immunol.

[6] He B, Raab-Traub N, Casali P, Cerutti A (2003). EBV-encoded latent membrane protein 1 cooperates with BAFF/BLyS and APRIL to induce T cell-independent Ig heavy chain class switching. J Immunol.

[7] Croia C, Serafini B, Bombardieri M, Kelly S, Humby F, Severa M (2013). Epstein-Barr virus persistence and infection of autoreactive plasma cells in synovial lymphoid structures in rheumatoid arthritis. Ann Rheum Dis.

[8] Kerkman PF, Fabre E, van der Voort EI, Zaldumbide A, Rombouts Y, Rispens T (2016). Identification and characterisation of citrullinated antigen-specific B cells in peripheral blood of patients with rheumatoid arthritis. Ann Rheum Dis.

[9] Kissel T, Reijm S, Slot LM, Cavallari M, Wortel CM, Vergroesen RD (2020). Antibodies and B cells recognising citrullinated proteins display a broad cross-reactivity towards other post-translational modifications. Ann Rheum Dis.

[10] Kraal LJN, Nijland ML, Germar KL, Baeten DLP, Ten Berge IJM, Fehres CM (2018). Anti-citrullinated protein antibody response after primary EBV infection in kidney transplant patients. PLoS One.

[11] Stevens SJC, Vervoort MBHJ, van den Brule AJC, Meenhorst PL, Meijer CJLM, Middeldorp JM (1999). Monitoring of Epstein-Barr Virus DNA Load in Peripheral Blood by Quantitative Competitive PCR. J Clin Microbiol.

[12] Balandraud N, Meynard JB, Auger I, Sovran H, Mugnier B, Reviron D (2003). Epstein-Barr virus load in the peripheral blood of patients with rheumatoid arthritis: accurate quantification using real-time polymerase chain reaction. Arthritis Rheum.

[13] Westergaard MW, Draborg AH, Troelsen L, Jacobsen S, Houen G (2015). Isotypes of Epstein-Barr virus antibodies in rheumatoid arthritis: association with rheumatoid factors and citrulline-dependent antibodies. Biomed Res Int.

[14] Baboonian C, Venables PJW, Williams DG, Williams RO, Maini RN (1991). Cross reaction of antibodies to a glycine/alanine repeat sequence of Epstein-Barr virus nuclear antigen-1 with collagen, cytokeratin, and actin. Ann Rheum Dis.

[15] Cornillet M, Verrouil E, Cantagrel A, Serre G, Nogueira L (2015). In ACPA-positive RA patients, antibodies to EBNA35-58Cit, a citrullinated peptide from the Epstein-Barr nuclear antigen-1, strongly cross-react with the peptide beta60-74Cit which bears the immunodominant epitope of citrullinated fibrin. Immunol Res.

[16] Fanelli I, Rovero P, Hansen PR, Frederiksen JL, Houen G, Trier NH (2022). Reactivity of Rheumatoid Arthritis-Associated Citrulline-Dependent Antibodies to Epstein-Barr Virus Nuclear Antigen1-3. Antibodies.

[17] Lanz TV, Brewer RC, Ho PP, Moon JS, Jude KM, Fernandez D (2022). Clonally expanded B cells in multiple sclerosis bind EBV EBNA1 and GlialCAM. Nature..

