# Supplementary figures and images for: Absence of Epstein-Barr virus DNA in anti-citrullinated protein antibody-expressing B cells of patients with rheumatoid arthritis
Source: Arthritis Res Ther. 2022 Oct 13;24:230. doi: 10.1186/s13075-022-02919-2 (PMC9559001; doi:10.1186/s13075-022-02919-2)

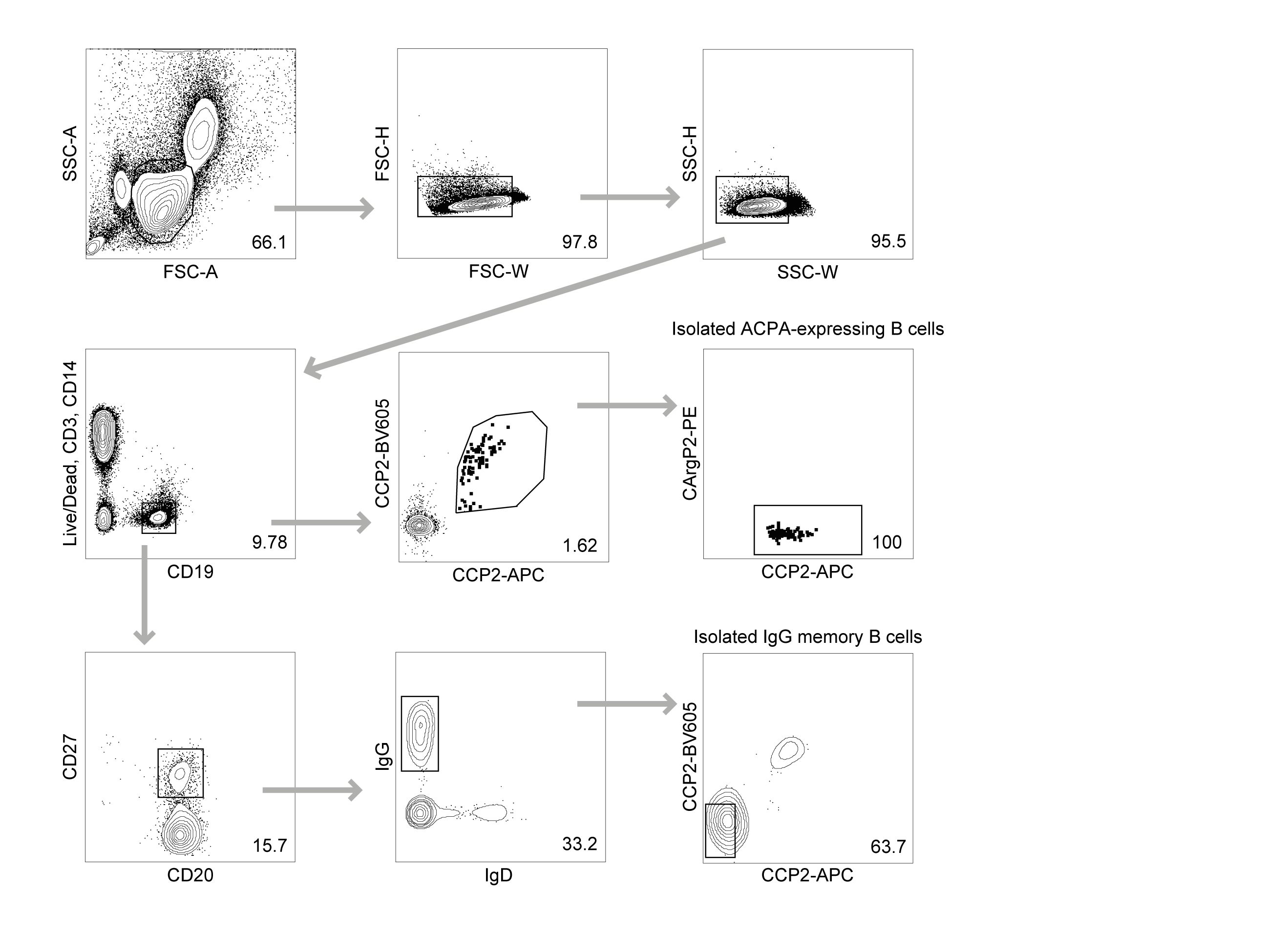

Supplement: Supplementary file 1 — Additional file 1: Supplementary figure 1. Gating strategy of the isolated ACPA-expressing and IgG memory B cell pools. [file 13075_2022_2919_MOESM1_ESM.tif]

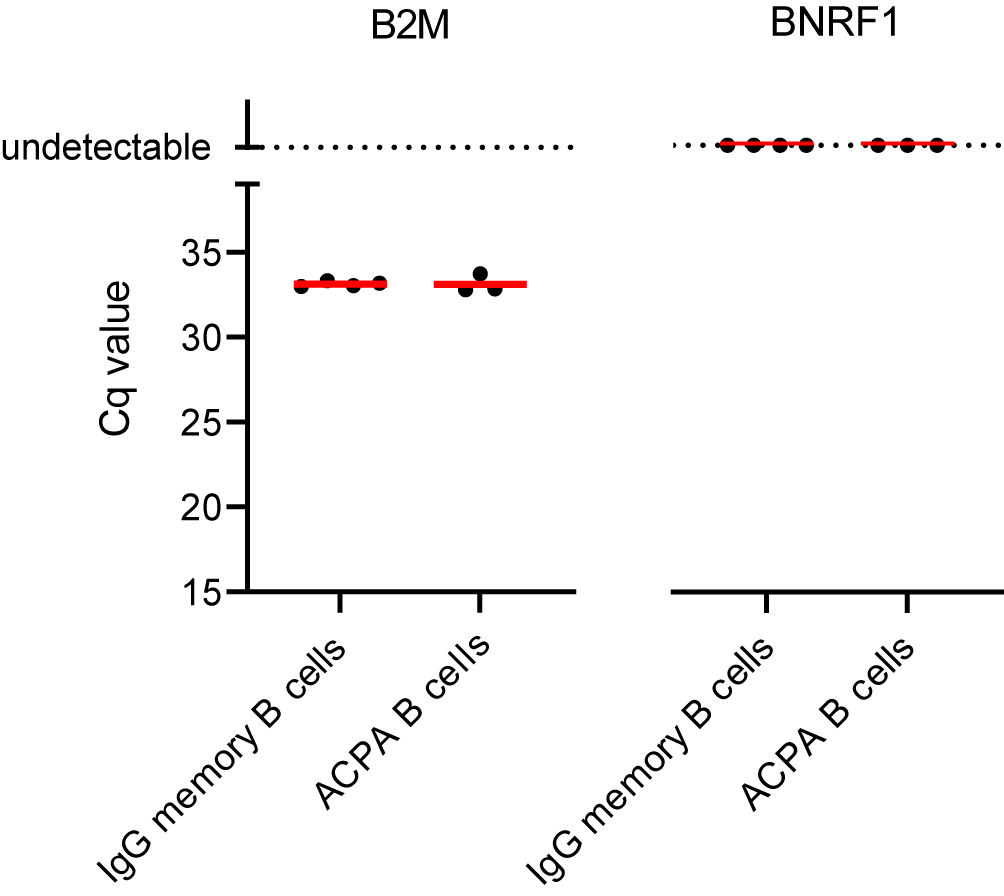

Supplement: Supplementary file 2 — Additional file 2: Supplementary figure 2. BNRF1 is undetectable in SF antigen-specific ACPA-expressing B cells. Left: B2M copies in DNA of SF IgG memory B cells and ACPA-expressing B cells as detected by qPCR. Right: no detection of BNRF1 by qPCR in DNA of SF IgG memory B cells, ACPA-expressing B cells and CD40L feeder cells only. Each dot represents a pool of 20 cells isolated directly into lysis buffer, from one donor. qPCR experiments were performed twice with similar outcomes. [file 13075_2022_2919_MOESM2_ESM.tif]
